# Physical activity, musculoskeletal disorders, burnout, and work engagement: a cross-sectional study on Italian white-collar employees

**DOI:** 10.3389/fpubh.2024.1375817

**Published:** 2024-04-30

**Authors:** Stefano Amatori, Erica Gobbi, Davide Sisti, Giorgia Pivato, Germana Giombini, Rosalba Rombaldoni, Giorgio Calcagnini, Marco B. L. Rocchi, Fabrizio Perroni

**Affiliations:** ^1^Department of Biomolecular Sciences, Division of Exercise and Health Sciences, University of Urbino Carlo Bo, Urbino, Italy; ^2^Department of Biomolecular Sciences, Service of Biostatistics, University of Urbino Carlo Bo, Urbino, Italy; ^3^Department of Economics, Social Science, and Politics, University of Urbino Carlo Bo, Urbino, Italy

**Keywords:** mental health, physical activity, well-being, workplace, Occupational Health (MeSH)

## Abstract

**Introduction:**

Both mental and physical health of office workers had a positive relationship with their work engagement, with the latter relationship being driven by the association of a healthy diet and physical activity (PA). This observational study aimed to investigate the associations between PA levels, musculoskeletal disorders (MSDs), burnout, and work engagement, in a sample of white-collar employees.

**Methods:**

A total of 615 workers (age 42.2 ± 9.5 years) successfully completed an online questionnaire comprising work-related information and standardized questionnaires on PA, MSDs, burnout and work engagement.

**Results:**

36.9% of the participants did not meet the PA guidelines, 19.0% adhered to them, and 44.1% exceeded them. A significant portion of participants reported suffering from MSDs, primarily neck/shoulder and/or low back/hip pain. Those exceeding PA guidelines had fewer MSDs compared to non-compliant participants and exhibited better mental health and work engagement. Compliance with PA guidelines was associated with a 38% reduced risk of emotional exhaustion, with an even greater reduction of 47% among those surpassing the guidelines.

**Discussion:**

PA could exert a positive effect on physical and mental health of employees, but only if performed above a certain amount. The study supports the need to identify workplaces as suitable for health-focused interventions and lifestyle changes.

## 1 Introduction

According to the recent report “Occupational Safety and Health in Europe: State and Trends 2023” by the European Agency for Safety and Health at Work, mental health disorders, along with musculoskeletal disorders (MSDs), are the leading causes of absence from work (1). Health conditions of adult workers impose a greater societal burden than work accidents; estimates from the Organization for Economic Co-operation and Development and the European Commission place annual mental disorder costs at over €600 billion in the EU (1). Depression-related illnesses, cardiovascular diseases, and MSDs are the most expensive conditions affecting employees, leading to increased presenteeism, absenteeism, and compromised work productivity (2, 3). MSDs contribute significantly to lost working days in EU Member States: upper extremity and low back MSDs account for about 39% of occupational diseases, with associated costs ranging from 0.5 to 2% of the EU’s gross national product, contributing to a loss of productivity across various economic sectors worldwide (4).

Physical health risks stem from multiple sources: manual workers (blue-collar) exhibit higher accident rates, shorter life expectancy, and reduced job expectancy, but in contrast, administrative workers (white-collar), though better off in these aspects, report a less favorable health situation. Indeed, sedentary jobs, through persistent sitting, contribute to MSDs and other health issues, such as cardiovascular diseases and weight gain. The European Survey of Enterprises on New and Emerging Risks (5) data ranks prolonged sitting as the EU27’s second most frequently reported risk factor. Moreover, sedentary behavior has been shown to be associated with worse cognitive performance, affecting several physiological mechanisms associated with cognition and mental health (6–8). Mental health is affected by various working conditions (work environment, workload pressure, social support, …); according to the EU labor force survey 2020, about 45% percent of employees report exposure to factors adversely impacting mental well-being (9). Furthermore, poor mental health is often associated with employees burnout: according to the World Health Organization, it represents an occupational phenomenon characterized by a chronic imbalance between job demands and resources (for example, job autonomy and supportive work relationships) (10). The definition of burnout includes three dimensions: emotional exhaustion (feeling of being depleted of one’s physical and emotional resources), depersonalisation (or cynism, representing the interpersonal context dimension of burnout), and reduced personal accomplishment (feeling of incompetence, lack of work productivity and achievement) (11, 12). However, burnout (and in particular emotional exhaustion) appear to be mitigated by engaging in physical activity (PA): in fact, several studies have reported a negative relationship between burnout and PA, highlighting that cases of burnout were lower in those who practiced more PA, both in sedentary (13) and non-sedentary workers (14–16).

So far, many studies investigated the effects of PA practice (both conducted in and out the workplace setting) on workers’ physical and mental health, showing that PA could be effective in reducing MSDs (4, 17) and mental health problems (17, 18), improving physical fitness (19, 20) and likely - indirectly - work performance (21). However, to the authors’ knowledge, the mutual interactions among PA, physical and mental health, and workplace well-being have been less studied. Recently, both mental and physical health have been reported to be positively associated with work engagement, defined as “a positive psychological construct characterized by vigor, dedication, and absorption” (22). A cross-sectional study conducted in Finland in 2014 among over 700 female employees found that both mental and physical health had a positive relationship with work engagement, with the latter relationship being driven by the association of a healthy diet and PA (22). However, it’s important to note that this study categorized PA as a binary variable, indicating whether participants met the American Heart Association’s PA guidelines or not. Consequently, the study could not determine whether participants just met these guidelines, exceeded them (and if so, by how much), or if higher levels of PA would have yielded greater benefits. Given the above, this observational study aimed to investigate the associations between PA levels (categorized as not reaching, reaching, or overcoming PA guidelines), MSDs, burnout, and work engagement, in a sample of Italian white-collar employees.

## 2 Materials and methods

### 2.1 Study design

The study has a cross-sectional observational design and has been approved by the Ethics Committee for Human Experimentation at the University of Urbino (approval number 20/2019). The paper’s structure follows the STROBE checklist (Strengthening the Reporting of Observational Studies in Epidemiology).

### 2.2 Setting

Data collection lasted approximately 4 months, from April to July 2022. The online questionnaire, developed using Google Forms, was distributed to participating companies via email. Upon receipt, employees were given a 15-day window to complete the questionnaire anonymously. The questionnaire was sent via email to the owners of each company, who then forwarded it to their employees. It was not possible to directly send the questionnaire to the respondents due to privacy maintenance reasons. Responses were recorded and subsequently imported into Microsoft Excel for analysis and processing.

### 2.3 Participants

Some companies located in the Italian territory, particularly in central Italy, from the manufacturing/industrial sector were contacted via email and invited to take part in the study. Presenting the research project, companies were informed about the possibility of receiving a personalized report containing the employees’ results at the end of the research. Upon acceptance, participating companies were requested to motivate their employees to complete the questionnaire to maximize response rates and obtain a comprehensive understanding of the company’s situation. To be eligible for participation, employees were required to meet the following criteria: have a good comprehension of the Italian language and fall within the working-age population (between 18 and 67 years old). Furthermore, participants were limited to office (white-collar) workers. The study involved the participation of approximately one thousand employees.

### 2.4 Measures

The questionnaire consisted of three different sections.

#### 2.4.1 General information and physical activity

In the first section, general information was collected, including anthropometric (height, weight) and sociodemographic characteristics and work-related details (length of employment within the company, presence, quantity, and duration of breaks during work, amount of time spent standing or sitting during a usual working day). Additionally, this section also requested information about leisure time PA performed by the employees. Employees were asked to specify the type of activity and the amount of time per week dedicated to each activity. Each activity was then converted into metabolic equivalents (METs) using the Compendium of Physical Activities (23) in order to compare the PA level of the employees with the World Health Organization’s (WHO) PA guidelines for adults (between 150 and 300 min of moderate-intensity aerobic PA or between 75 and 150 min of vigorous-intensity aerobic PA, or an equivalent combination of moderate-and vigorous-intensity activity, throughout the week) (24). Participants were then categorized into: not respecting, respecting, or overcoming the PA guidelines; these three groups were used for all the statistical analyses.

#### 2.4.2 Musculoskeletal disorders

A subsequent part of the questionnaire was dedicated to the Cornell Musculoskeletal Discomfort Questionnaire (CMDQ) for sedentary workers. This self-reported questionnaire measures musculoskeletal discomfort in 11 body segments by assessing the frequency (number of times per week) of experiencing pain, the level of discomfort caused by the pain, and the extent to which it interferes with work capacity (25). The results of this questionnaire can be interpreted by simply counting the number of symptoms per person, or by calculating a total score. A single score for each component is calculated first: frequency (0 = never, 1.5 = 1/2 times/week, 3.5 = 3/4 times/week, 5 = every day); discomfort (1 = slightly uncomfortable, 2 = moderately uncomfortable, 3 = very uncomfortable); interference (1 = not at all, 2 = slightly interfered, 3 = substantially interfered). The total score is then calculated by multiplying the above scores (frequency × discomfort × interference, range 0–45). In order to allow a more straightforward interpretation of the data, a correlation matrix was used to investigate the mutual associations between body segments, and accordingly, it was decided to reduce the number of body parts to four: (1) neck, shoulders, and upper back, (2) upper arms, forearms, and wrists, (3) lower back and hip, (4) thighs, knees, and lower legs.

#### 2.4.3 Work engagement and burnout

The third part of the questionnaire aimed to investigate how employees felt at work. For this purpose, two questionnaires were used: the Utrecht Work Engagement Scale short form (UWES-9) and the Maslach Burnout Inventory (MBI). The UWES-9 questionnaire assesses work engagement, which refers to the level of involvement, satisfaction, dedication, vigor, and absorption experienced by employees. Work engagement is characterized by high energy levels and strong identification with one’s work. The UWES-9 questionnaire consists of 9 items divided into three subscales: vigor, dedication, and absorption (26, 27). Responses to the questionnaire were rated on a 7-point Likert scale (0 = never, 6 = every day) (28). The UWES-9 total score is calculated by averaging the points of each item, where the higher the score is, the higher the work engagement. The MBI is a multidimensional questionnaire consisting of 22 items that assess three independent dimensions of burnout syndrome (29, 30): emotional exhaustion (or exhaustion), depersonalisation (or cynicism), and personal accomplishment (or professional efficacy) (31). The scores are calculated by summing the items pertaining to each dimension. According to Leiter and Maslach (32), cut-offs values for the MBI were calculated using standardized values (z-scores), defining the following boundaries: high exhaustion at *z = Mean + (SD × 0.5)*, high depersonalisation at *z = Mean + (SD × 1.25)*, and high personal accomplishment at *z = Mean + (SD × 0.10)*. The critical boundaries then depend on the specific group’s population norms.

### 2.5 Statistical analyses

Data are presented as counts, relative frequencies, mean ± standard deviation, and median [first and third quartiles] were appropriate. An independent-sample Kruskal-Wallis H test was used to compare the severity of MSD between PA groups, and pairwise comparisons with Bonferroni correction were also conducted. The non-parametric test was chosen as the MSD responses did not have a normal distribution. A one-way MANOVA was used to test the differences in the responses to the UWES-9 and MBI questionnaires among the three PA groups; LSD post-hoc tests were conducted to explore pairwise differences between the PA groups. Finally, logistic regression was performed (best subset selection method, with the Akaike Information Criterion employed as the criterion for model selection) to test the effect of some predictor variables (age, gender, BMI, presence of MSD, and PA group) on the three subscales of MBI, considered as binary variables (below or above the cut-off boundaries). The strength of association was quantified as odds-ratio (OR) with relative confidence intervals; moreover, the area under the curve (AUC) was used to quantify the overall goodness of classification, and Nagelkerke R^2^ the goodness of fitting of the model. All the analyses were performed with SPSS v.26 (IBM, Armonk, NY, USA) and RStudio v.2023 (Posit Software, PBC), at a standard significance level of alpha = 0.05.

## 3 Results

### 3.1 Participants characteristics

Approximately one-thousand employees were contacted; of them, 615 (452 males, 163 females; age: 42.2 ± 9.5; height: 174.9 ± 8.3 cm; weight: 75.1 ± 14.0; BMI: 24.4 ± 3.5 kg/m^2^) successfully completed the questionnaire. 468 participants (76.1%) declared practicing PA in their leisure time, with a median frequency of 4 [2.50–6] times per week and a total time of 240 [150–360] minutes per week. As described in the methods section, METs for each activity were derived from the Compendium of Physical Activities (23), and participants were categorized according to the WHO’s PA guidelines as follows: 227 (36.9%) did not respect, 177 (19.0%) did respect, and 271 (44.1%) did overcome the PA guidelines. The participants’ work-related characteristics are reported in Table 1. Most participants had a “traditional” 5-day–8 h/day – work week, spending most of their time (≈80%) in the seated position. Almost 90% of the participants reported having breaks during work hours, with an average of two 8-min breaks per day.

**Table 1:** Work-related characteristics.

	Frequency (%)	Median [5th–95th percentile]
Working days/week	–	5 [5–5]
Breaks (yes)	542 (88.1%)	–
Breaks number	–	2 [1–3]
Breaks duration (min)	–	10 [5–15]
Standing time (h)	–	1 [0–4]
Seated time (h)	–	7 [4–8]
Comfort workstation (yes)	462 (75.1%)	–

### 3.2 Associations between PA and MSDs

**Figure 1:**
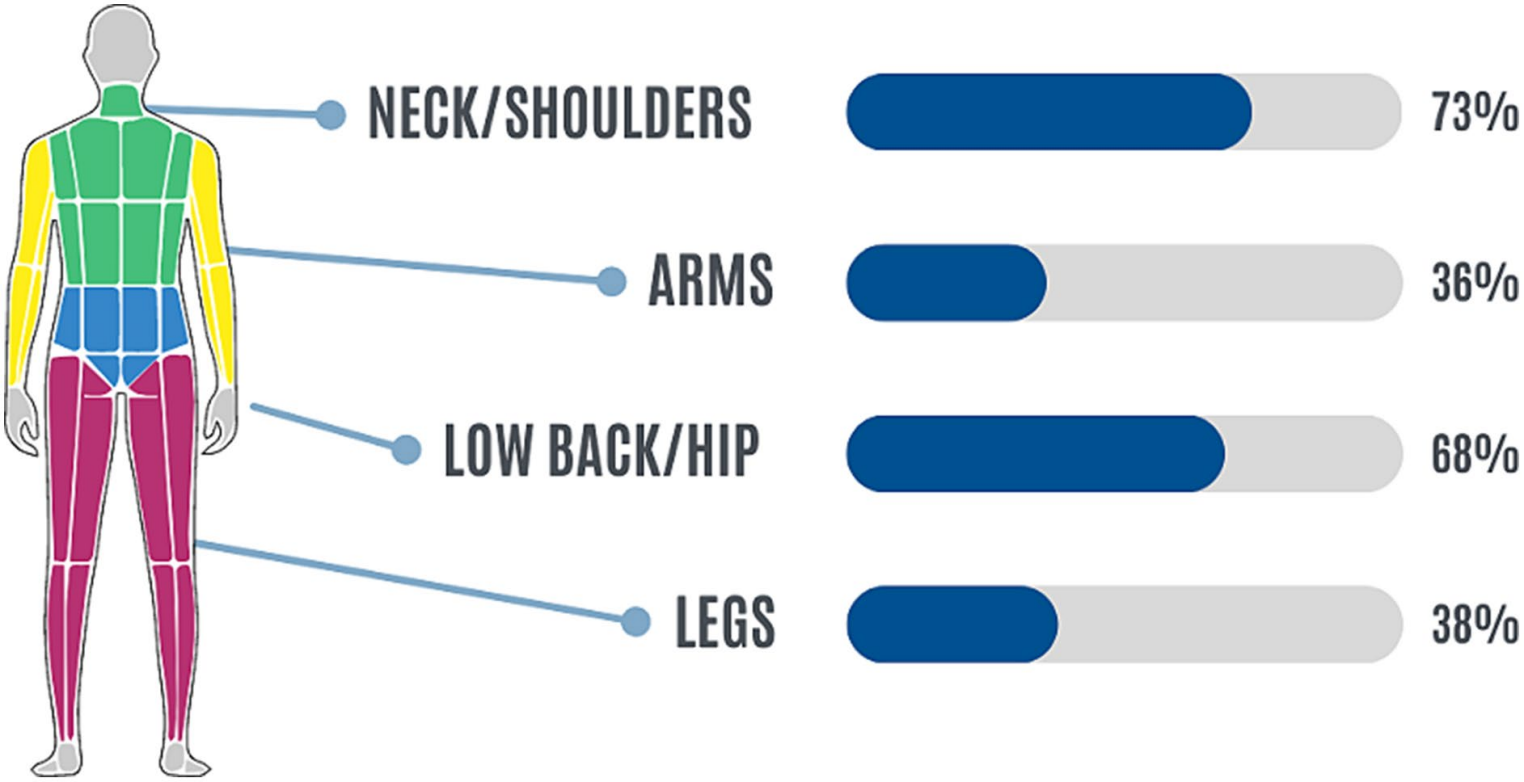
Relative frequencies of MSDs in the four body districts: neck/shoulders/upper back (green), arms (yellow), low back/hip (blue), and legs (purple).

A high proportion of participants reported suffering from MSDs. In particular, about 70% of them reported having neck/shoulders and/or low back/hip pain at least 1–2 times per week. Figure 1 represents the relative frequencies of MSDs in the four body districts.

**Figure 2:**
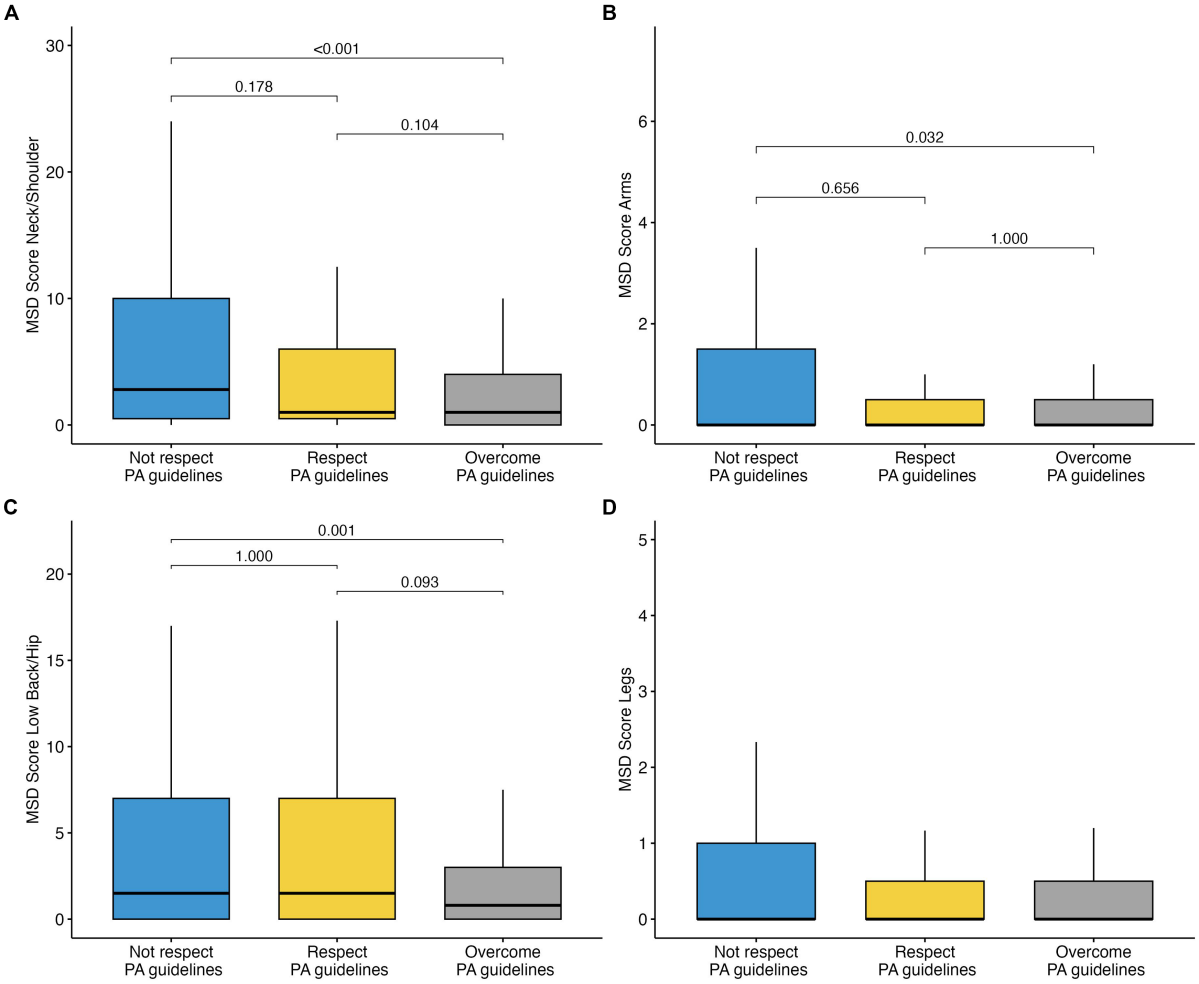
Boxplots of MSD among the three PA groups for the four body districts: **(A)** neck/shoulders; **(B)** arms; **(C)** low back/hip; **(D)** legs. Bonferroni-adjusted *p*-values of pairwise comparisons are also reported, except for panel **(D)**, in which multiple comparisons were not performed because the overall test did not show significant differences across groups. Outliers are not represented in the plots due to graphical reasons.

The MSD score (frequency × discomfort × interference) was then used to compare the distribution of MSD among the three groups of PA (not respecting, respecting, overcoming guidelines) (Figure 2). Results showed a positive effect of PA for all body districts except for legs. In particular, for the neck/shoulder region, those who overcame the PA guidelines showed fewer MSD than those who did not respect them (median = 1.0 [0–4] vs. 2.5 [0.5–11.3], *H* = 78.5, *p* < 0.001). At the same time, no significant differences were reported between those who overcame and who respected (*p* = 0.104) and who respected and did not the guidelines (*p* = 0.178). Also, for arms and low back, the same significant comparisons were detected, with the overcoming group reporting lower MSD scores than those who did not respect the guidelines (arms: median = 0 [0–0.5] vs. 0 [0–1.92], *H* = 34.1, *p* = 0.032; low back: median = 0.8 [0–3] vs. 1.5 [0–7], *H* = 58.1, *p* = 0.001). These results suggest that reaching the PA guidelines does not seem enough to have a significant protective effect against MSD.

### 3.3 Associations between PA, burnout, and work engagement

A one-way MANOVA was conducted to test the differences in UWES-9 and MBI responses between the three PA groups. The main results showed a significant effect of PA practice on all the analysed subscales, except for UWES-9 Absorption (*p* = 0.371) and MBI Depersonalisation (*p* = 0.575). When looking at the post-hoc tests, it can be noticed how the “not-respect” and “respect” groups did not show any significant differences (except for MBI Emotional Exhaustion), while the “overcome” group reported significantly better results in all the subscales (except for those that were not significant in the global test). Notably, in the MBI Personal Accomplishment subscale, the “overcome” group showed significantly better results also compared with the “respect” group (*p* = 0.006). These results suggest that PA could exert a positive effect on the mental health of employees, but only if performed above a certain amount. Data are reported in Table 2.

**Table 2:** UWES-9 and MBI scores (Mean ± SD), grouped according to PA groups.

	Not-respect	Respect	Overcome	*p*(F)
UWES-9				
Vigor	4.08 ± 1.58	4.34 ± 1.42	4.59 ± 1.30^a^	**<0.001**
Dedication	4.02 ± 1.77	4.27 ± 1.59	4.54 ± 1.57^a^	**0.002**
Absorption	4.77 ± 1.25	4.79 ± 1.25	4.91 ± 1.15	0.371
Total	4.29 ± 1.35	4.47 ± 1.25	4.68 ± 1.20^a^	**0.003**
MBI				
Emotional exhaustion	25.57 ± 11.27	22.05 ± 10.68^a^	21.37 ± 9.44^a^	**<0.001**
Depersonalisation	8.93 ± 5.01	8.74 ± 4.45	8.49 ± 4.62	0.575
Personal accomplishment	34.33 ± 7.37	34.11 ± 7.08	36.25 ± 6.75^a,b^	**0.002**

UWES-9, Utrecht Work Engagement Scale-9; MBI, Maslach Burnout Inventory; ^a^significantly different from “not-respect,” ^b^significantly different from “respect”. Bold values in table highlight statistical significance.

MBI subscales can also be interpreted considering the cut-off values, for each subscale. When examining the proportion of subjects who scored above the cut-off values, results showed that 173 participants (28.1%) had EE, 86 participants (14.0%) had DE, and 314 participants (51.1%) had PA values above the cut-off limits, showing almost one-third of participants with concerns of emotional exhaustion and only half of them reporting favorable scores of personal accomplishment.

Then, three logistic regressions were used in order to explore the impact of some predictor variables on the MBI subscales: age, gender, BMI, presence of MSD, and PA group were considered as predictors. The three models (on EE, DE, and PA, respectively) showed fair goodness of classification, with AUC values of 0.67 and 0.68 for the first two models and 0.59 for the latter. The goodness of fitting (Nagelkerke R^2^) was 0.101, 0.086, and 0.038, respectively. Results are reported in Table 3.

**Table 3:** Results of the logistic regressions: coefficients estimate with standard errors (SE), *p*-values, and odds-ratios [95% confidence intervals] are reported for the variables which entered in each model.

	Model 1: emotional exhaustion			Model 2: depersonalisation			Model 3: personal accomplishment		
	Coefficient (SE)	*p*-value	Odds-ratio [95% CI]	Coefficient (SE)	*p*-value	Odds-ratio [95% CI]	Coefficient (SE)	*p*-value	Odds-ratio [95% CI]
Age	–	–	–	−0.04 (0.01)	0.002	0.961 [0.937–0.986]	0.02 (0.01)	0.009	1.023 [1.006–1.041]
Gender = F	–	–	–	−0.82 (0.32)	0.010	0.442 [0.238–0.821]	–	–	–
BMI	–	–	–	–	–	–	–	–	–
PA respect	−0.48 (0.26)	0.065	0.621 [0.374–1.031]	−0.41 (0.34)	0.231	0.662 [0.337–1.301]	–	–	–
PA overcome	−0.64 (0.21)	0.002	0.528 [0.352–0.793]	−0.53 (0.27)	0.051	0.590 [0.347–1.002]	–	–	–
MSD neck	0.57 (0.25)	0.021	1.770 [1.090–2.875]	–	–	–	−0.46 (0.19)	0.016	0.631 [0.433–0.919]
MSD arms	0.38 (0.19)	0.055	1.456 [0.992–2.138]	0.35 (0.25)	0.151	1.424 [0.879–2.306]	–	–	–
MSD low back	–	–	–	–	–	–	–	–	–
MSD legs	0.62 (0.19)	0.001	1.853 [1.269–2.706]	0.68 (0.24)	0.005	1.980 [1.226–3.196]	−0.26 (0.17)	0.130	0.768 [0.546–1.080]

F = female; BMI = body mass index; PA = Physical activity; MSD = musculoskeletal disorders.

When looking at model 1 (emotional exhaustion), PA and MSD entered the model, with the former exerting a protective effect against emotional exhaustion, while the latter having a detrimental effect; it can be noticed how participants respecting the PA guidelines had a −38% risk of suffering of emotional exhaustion, and in the participants who overcome the PA guidelines, the risk decreased to −47%. Approximately the same results were obtained by model 2 (depersonalisation), with age and gender entering the model as well: indeed, it seemed that each one-year decrease in age was associated with a 4% decrease in the odds of developing depersonalisation issues (OR: 0.96), and being female was associated with a 56% lower odds of developing depersonalisation issues (OR: 0.44). Lastly, the third model (personal accomplishment) showed a positive effect of age, and a negative effect of neck/shoulder and legs MSD on personal accomplishment levels; however, the third model (personal accomplishment) had limited goodness of fitting (*R*^2^ = 0.038), suggesting that other unexplored factors may play a more substantial role in determining personal accomplishment outcomes.

## 4 Discussion

This cross-sectional study aimed to investigate the associations between PA levels and the occurrence of MSDs, burnout, and work engagement among Italian white-collar employees. The main findings suggest that leisure-time PA might play a pivotal role in reducing the severity of musculoskeletal problems, enhancing work engagement, and protecting from the development of burnout. Notably, our study reveals that merely reaching PA guidelines may not be sufficient to achieve significant positive effects on the measured parameters. Indeed, a few studies (15, 33) investigated the leisure-time PA dividing the participants into who met and who did not the guidelines, and found significantly lower levels of burnout in those who met the guidelines, but no significant effects on MSDs. However, these studies did not investigate whether higher levels of PA (i.e., overcoming the PA guidelines) would provide greater beneficial effects. On this line, Jonsdottir and colleagues (16) reported that light PA practice (defined as walking/cycling to work or gardening for 2 h/week) was sufficient to exert a positive effect on stress and burnout levels, but they also found that moderate-to-vigorous PA practice had a stronger effect, further lowering the relative risks ratios. Overall, these findings are coherent with the results of the present study, as in our sample, participants who met the PA guidelines did not show significantly lower MSDs or work-engagement levels, but only lower levels of emotional exhaustion; in contrast, workers who overcame the PA guidelines, showed significantly lower MSDs, lower emotional exhaustion, higher personal accomplishment, and better work engagement, suggesting that limiting oneself to reaching the guidelines does not seem to be enough to top up the benefits. Furthermore, we found that having MSDs increased the risk of developing burnout symptoms; this is in line with the results by Biddle et al. (33), who also reported that a large proportion of general practitioners experiencing burnout also had neck, shoulder, or back pain. Biddle and colleagues also reported that most of their participants did not meet the PA guidelines and spent most of their working time in prolonged sitting bouts; interestingly, those who had more regular breaks from prolonged sitting showed lower burnout and greater psychological well-being than those who had fewer breaks. Participants of our sample reported a “standard” level of breaks during the work time, with a median of two 8-min pauses, in line with the Italian National regulation, which guarantees a minimum of one 10-min break for every 6 hours of work. Notably, a recent German study reported that employees frequently skipped and/or interrupted their work breaks, and this was related to physical and mental health complaints (34).

In our study, and in accordance with other evidence (13, 15), the emotional exhaustion component of burnout was the one that seemed more influenced by the PA practice. A possible explanation, as reported by Aronsson et al. (11), is that burnout is a process in time, meaning that there is a time lag between the development of the three components. Indeed, emotional exhaustion, due to the high coping efforts with external demands, acts as a trigger for depersonalisation, which in turn causes a reduction in personal accomplishment, and this is a vicious cycle as low gratification contributes to further emotional exhaustion, and so on. This time effect is of pivotal importance, as persons who show high emotional exhaustion scores might not have still developed symptoms of depersonalisation or low personal accomplishment, and the fact that PA practice seems to have a greater influence on the first component of burnout could be a promising result, as it could means that - potentially - reducing the emotional exhaustion by PA practice would lead to a prevention of the other burnout dimensions.

Veromaa et al. (22) conducted a cross-sectional study in Finland aimed to investigate the factors influencing work engagement in a sample of female employees, and after dividing the sample into who met and did not meet the PA guidelines, found that the former showed higher levels of work engagement. This partially agrees with our results, as we found out that only the group who overcame the PA guidelines showed significantly higher levels of work engagement, although a smaller non-significant difference was also observed for whom reached the guidelines. This data suggests that merely reaching the guidelines could not be enough to improve work engagement, but higher PA levels, likely at moderate-to-vigorous intensity, are needed. Increasing the levels of work engagement should be of particular importance for both employers and employees, as it has been shown that it is strictly related to job performance. However, this relationship is not linear but inverted U-shaped, indicating that work performance is not only influenced by the presence of good working conditions, but also closely related to the state of each individual (35).

The findings of this study contribute to the existing literature by providing further support and highlighting the crucial role of engaging in PA in promoting the overall health of workers, both in physical and mental dimensions. What this study adds is the idea that the quantity and quality (frequency, duration, and intensity) of PA undertaken may play a pivotal role in the observed beneficial effects for employees. Given the substantial amount of time individuals spend at their workplaces, this context has been proposed as a suitable environment for implementing interventions aimed at fostering workers’ health through lifestyle modifications. Various types of physical activities (stretching, strength training, aerobic exercises, high-intensity interval training, or various combinations thereof) have been proposed, yielding promising but occasionally divergent outcomes (4, 19, 20, 36, 37). Well-designed, randomized, and controlled studies remain essential to explore the impact of PA on workers’ physical and mental well-being. Furthermore, future research should focus on quantifying the economic implications of these interventions for the companies themselves, to further encourage and raise employers’ awareness about the significance of their employees’ health.

Several limitations should be considered when interpreting the findings of this study. First, the cross-sectional design of the study restricts our ability to establish causality, as we can only examine biunivocal associations at a single point in time, cautioning against over-interpreting the causal relationship between PA and health outcomes. Additionally, the measurement of PA relied on self-assessment, which is susceptible to social desirability bias, potentially leading to overestimating PA levels. Moreover, the exact participation rate in the intervention group cannot be ascertained, as we lack information on how many employees received and read the email invitation, introducing a degree of uncertainty in the overall participation rate. Another limitation that should be acknowledged is the generalizability of our findings beyond the Italian white-collar workers. While our sample provides valuable insights into this population, it is essential to recognize that cultural and demographic factors may influence the relationship between PA and health outcomes differently in other populations or cultural contexts. Furthermore, our study lacks a formal economic analysis of the potential implications of PA interventions. While we recognize the importance of considering economic factors in public health interventions, our study did not include a comprehensive economic evaluation. This limitation highlights an important area for future research, as incorporating economic analyses could provide valuable insights into the cost-effectiveness and feasibility of PA interventions. In light of these limitations, future research should focus on employing more precise and objective measurements of PA, such as wearable devices or activity trackers, to enhance the accuracy of the collected data. Furthermore, investigating longitudinal study designs would enable a better understanding of the temporal relationships between PA, burnout, and other relevant variables, providing more robust evidence for potential causal relationships. Lastly, future studies should aim to include formal economic evaluations to better inform decision-making and policy development in this area.

## 5 Conclusion

Our cross-sectional study on white-collar employees highlights the pivotal role of leisure-time PA in reducing musculoskeletal problems, enhancing work engagement, and preventing burnout, echoing principles of sports medicine. Our findings challenge the adequacy of meeting PA guidelines, aligning with research emphasizing the need to surpass these standards (16, 33). Particularly, our focus on emotional exhaustion aligns with burnout research (13, 15), hinting at a time-sensitive cascade effect (11). Our work extends Veromaa et al.’s (22) findings on work engagement, emphasizing the value of PA beyond guidelines. Enhanced work engagement, with its nonlinear impact on job performance (35), underscores the importance of personalized well-being strategies. Integrating these insights into workplaces through tailored PA initiatives holds promise for employee health. Future research should explore economic implications and employ precise PA measurement methods for deeper understanding. In conclusion, our study bridges sports medicine and workplace wellness, accentuating the impact of PA on workers’ health. It emphasizes the necessity of exceeding PA guidelines for optimal benefits and offers actionable insights for healthier, more engaged, and less burnout-prone employees.

## Data availability statement

The raw data supporting the conclusions of this article will be made available by the authors, without undue reservation.

## Ethics statement

The studies involving humans were approved by Ethics Committee for Human Experimentation at the University of Urbino (approval number 20/2019). The studies were conducted in accordance with the local legislation and institutional requirements. The participants provided their written informed consent to participate in this study.

## Author contributions

SA: Conceptualization, Investigation, Visualization, Writing – original draft, Writing – review & editing. EG: Conceptualization, Investigation, Writing – original draft, Writing – review & editing. DS: Data curation, Formal analysis, Methodology, Writing – original draft, Writing – review & editing. GP: Data curation, Writing – original draft, Writing – review & editing. GG: Project administration, Writing – original draft, Writing – review & editing. RR: Project administration, Writing – original draft, Writing – review & editing. GC: Project administration, Writing – original draft, Writing – review & editing. MR: Formal analysis, Methodology, Supervision, Writing – original draft, Writing – review & editing. FP: Supervision, Writing – original draft, Writing – review & editing.
